# Effectiveness of KarXT (xanomeline-trospium) for cognitive impairment in schizophrenia: post hoc analyses from a randomised, double-blind, placebo-controlled phase 2 study

**DOI:** 10.1038/s41398-022-02254-9

**Published:** 2022-11-21

**Authors:** Colin Sauder, Luke A. Allen, Elizabeth Baker, Andrew C. Miller, Steven M. Paul, Stephen K. Brannan

**Affiliations:** 1 Karuna Therapeutics, Boston, MA USA; 2 Cambridge Cognition, Cambridge, UK

**Keywords:** Schizophrenia, Scientific community

## Abstract

The muscarinic receptor agonist xanomeline improved cognition in phase 2 trials in Alzheimer’s disease and schizophrenia. We present data on the effect of KarXT (xanomeline–trospium) on cognition in schizophrenia from the 5-week, randomised, double-blind, placebo-controlled EMERGENT-1 trial (NCT03697252). Analyses included 125 patients with computerised Cogstate Brief Battery (CBB) subtest scores at baseline and endpoint. A post hoc subgroup analysis evaluated the effects of KarXT on cognitive performance in patients with or without clinically meaningful cognitive impairment at baseline, and a separate outlier analysis excluded patients with excessive intraindividual variability (IIV) across cognitive subdomains. ANCOVA models assessed treatment effects for completers and impairment subgroups, with or without removal of outliers. Sample-wide, cognitive improvement was numerically but not statistically greater with KarXT (*n* = 60) than placebo (*n* = 65), *p* = 0.16. However, post hoc analyses showed 65 patients did not exhibit clinically meaningful cognitive impairment at baseline, while eight patients had implausibly high IIV at one or both timepoints. Significant treatment effects were observed after removing outliers (KarXT *n* = 54, placebo *n* = 63; *p* = 0.04). Despite the small sample size, a robust (*d* = 0.50) and significant effect was observed among patients with cognitive impairment (KarXT *n* = 23, placebo *n* = 37; *p* = 0.03). These effects did not appear to be related to improvement in PANSS total scores (linear regression, *R*^*2* ^= 0.03). Collectively, these findings suggest that KarXT may have a separable and meaningful impact on cognition, particularly among patients with cognitive impairment.

## Introduction

Schizophrenia is characterised by a wide array of cognitive deficits in executive functioning, attention, memory, and processing speed, which often precede the onset of psychosis and are associated with substantial functional impairment [1]. Although not all patients with schizophrenia exhibit meaningful cognitive impairment, those who do have poorer interpersonal functioning and are less likely to live independently and be employed [2, 3]. Unfortunately, there are currently no approved treatments for cognitive impairment associated with schizophrenia (CIAS) [4]. Additionally, drug development programmes specifically targeting CIAS have suffered from inconsistent findings, and to date no drug candidates have proven efficacious. Thus, there remains an unmet need for CIAS-specific treatments as well as for the development of antipsychotic treatments that can meaningfully impact CIAS.

KarXT (xanomeline–trospium; Karuna Therapeutics™, Boston, MA, USA) is an investigational treatment that has shown early promise in the treatment of positive and negative symptoms of schizophrenia [5]. Unlike all currently approved treatments for schizophrenia, KarXT does not directly bind to dopamine receptors; instead, the therapeutic effects of KarXT appear to be mediated through direct agonism of muscarinic acetylcholine receptors (mAChRs) [6, 7]. Robust preclinical and clinical evidence shows that mAChRs are crucially involved in learning and memory circuits and significant efforts have been made to develop drugs that directly or indirectly stimulate mAChRs in order to treat cognitive impairment [8, 9]. Substantial pharmacologic evidence exists demonstrating that mAChR agonists have robust efficacy in reversing cognitive impairment in preclinical models relevant to Alzheimer’s disease (AD) and schizophrenia [8, 10–12]. Thus, although KarXT is currently under development for its potential antipsychotic properties, there is a high likelihood that it may also exhibit procognitive effects.

KarXT combines the M_1_/M_4_-preferring muscarinic receptor agonist xanomeline with trospium, a U.S. Food and Drug Administration–approved peripheral muscarinic receptor antagonist that does not cross the blood-brain barrier [13]. KarXT was developed to mitigate the peripheral procholinergic side effects of xanomeline by combining it with the competing peripheral anticholinergic activity of trospium, with the aim of improving tolerability while still preserving procholinergic activity at M_1_/M_4_ muscarinic receptors in the central nervous system (CNS). Extensive preclinical literature suggests that M_1_ muscarinic receptor agonists can modulate CNS circuits associated with memory, learning, and attention [8, 14]. The potential impact of mAChR agonists on memory and attention is also supported by evidence from studies in healthy and cognitively impaired human subjects [15, 16]. Additionally, although the bulk of the evidence from preclinical and human studies suggests that selectively targeting the M_4_ muscarinic receptor may yield potent antipsychotic effects [5, 9, 17, 18], there is also evidence that M_4_ muscarinic receptors may have a role in regulating cognitive processes. Specifically, recent work suggests that selective M_4_ muscarinic receptor agonists could be useful in the treatment of cognitive deficits by restoring inhibitory tone in cognitive circuits [9, 14, 19, 20].

KarXT preferentially activates both M_1_ and M_4_ muscarinic receptors, suggesting the potential for both procognitive and antipsychotic treatment effects. Indeed, xanomeline has been shown to improve cognition in patients with AD [21] and in a small phase 2 proof-of-concept study in schizophrenia [22]. Recently, the phase 2 EMERGENT-1 study of KarXT in patients with schizophrenia demonstrated a marked antipsychotic effect with KarXT vs. placebo [5].

KarXT met the primary endpoint of change in Positive and Negative Syndrome Scale (PANSS) total score at week 5 (–17.4 points with KarXT vs. –5.9 points with placebo; least squares [LS] mean difference, –11.6 points; *p* < 0.001), as well as multiple secondary endpoints [5]. To further assess the potential benefits of KarXT in treating CIAS, a brief computerised battery was included as an exploratory endpoint in this study. Although not statistically significant, a preliminary analysis of these data suggested KarXT had a modest treatment effect (*d* = 0.20) [5]. However, the EMERGENT-1 trial design and study population were optimized for the trial’s primary objectives, which made it more difficult to assess the treatment effect on cognitive impairment and potentially resulted in an underestimation of the true treatment effect. Here we present a series of post hoc analyses of the exploratory cognitive endpoint in the phase 2 EMERGENT-1 study to account for the pitfalls inherent in using a computerised cognitive battery to assess cognitive performance in acutely psychotic patients with schizophrenia.

The main challenge to accurately assessing the effects of KarXT on CIAS pertains to the sample selection biases inherent in a study designed to assess the antipsychotic effects of KarXT in acutely psychotic individuals. Assessing cognition in patients with schizophrenia when patients are acutely psychotic can result in high rates of spurious test results that may not be reflective of true cognitive performance [23]. However, it is important to note that positive symptoms of schizophrenia are not necessarily strongly correlated with CIAS [1]. Thus, although the patient population in EMERGENT-1 clearly exhibited psychosis or positive symptoms, they may not necessarily exhibit significant cognitive impairments. Although most patients with schizophrenia exhibit at least some level of cognitive impairment compared with their premorbid state, there is a sizeable number of patients (approximately 30%) with schizophrenia who perform within the normal range on a variety of cognitive tests [1]. Furthermore, the requirements of participating in a 5-week long clinical trial may inadvertently select for patients with more intact cognitive functioning. In clinical trials, patients are required to complete assessments and procedures that are significantly more complex than those found in standard clinical care, placing a floor on the overall level of functioning necessary to participate. CIAS is strongly related to functional impairment [3] and, as such, patients selected for trials like the EMERGENT-1 study may artificially select for patients with higher overall levels of cognitive functioning. In turn, this may result in ceiling effects and other methodological pitfalls due to the varied nature of cognitive performance in schizophrenia.

Additionally, because the cognitive assessment in EMERGENT-1 was exploratory and not the focus of the study design, the testing battery and the conditions in which it was administered likely resulted in increased variability in testing performance. Performance on cognitive tests can be substantially impacted by factors such as the testing environment, time of day, fatigue, and effort, just to name a few [24, 25]. EMERGENT-1 was not designed to control such factors and, thus, a higher degree of intraindividual variability in performance is to be expected. Furthermore, computerised cognitive batteries, although benefiting from ease of use (i.e., a trained neuropsychologist is not needed for administration), have still been found to have greater variability in performance in certain circumstances, such as in the assessment of acutely psychotic participants [23]. These methodological challenges are likely to result in outliers that do not reflect true performance, lead to excessive error variance [26], substantially bias results toward the null, and which must be addressed to estimate the true treatment effect.

In order to better characterise the potential therapeutic effects of mAChR agonists like KarXT on CIAS, we present two post hoc analyses of data from the phase 2 EMERGENT-1 study. These analyses address the potential confounding factors highlighted above by (1) limiting analyses to patients who are experiencing clinically meaningful cognitive impairment and therefore are able to manifest a measurable benefit from treatment and (2) applying well-accepted and prespecified methodological approaches to exclude assessment data that are likely to be difficult to reliably interpret and unlikely to represent true cognitive performance.

## Patients and methods

### Study design and participants

EMERGENT-1 was a phase 2, randomised, double-blind, placebo-controlled, 5-week trial that assessed the efficacy and safety of KarXT in 182 adults 18 to 60 years of age experiencing an acute exacerbation of schizophrenia (ClinicalTrials.gov NCT03697252) [5]. Enrolment criteria and the study design and procedures have been described in detail previously [5]. Briefly, patients with a primary diagnosis of schizophrenia who were experiencing an acute exacerbation or relapse of psychosis requiring hospitalisation, as evidenced by baseline PANSS total score 80 or higher and a Clinical Global Impression-Severity scale score 4 or higher at screening and baseline, were enroled at 12 inpatient sites in the United States between September 2018 and August 2019. The study excluded patients with a primary disorder other than schizophrenia, history of treatment resistance to antipsychotics, or decrease in PANSS total score 20% or higher between screening and baseline. Eligible patients were randomly assigned in a 1:1 ratio to receive twice-daily dosing of oral KarXT or placebo. KarXT was dosed flexibly, starting with xanomeline 50 mg/trospium 20 mg twice daily and increasing to a maximum dose of 125 mg/30 mg twice daily. All patients provided written informed consent prior to initiation of study procedures or interventions, and the trial protocol was approved by a central institutional review board (Copernicus Group, Cary, NC, USA). The study protocol is available at https://www.nejm.org/doi/suppl/10.1056/NEJMoa2017015/suppl_file/nejmoa2017015_protocol.pdf.

### Assessments

At study baseline and end of study (week 5), patients completed a cognitive battery targeting multiple cognitive subdomains to evaluate the impact of KarXT on cognitive performance. Post hoc analyses focused on treatment differences in individuals with or without clinically meaningful cognitive impairment at study baseline and extreme variability in cognitive performance.

The Cogstate Brief Battery (CBB) [27] was administered to patients in EMERGENT-1 to target the core domains of CIAS in a low-burden manner. The CBB included five computerised cognitive tests, which were completed on an iPad. The tasks and cognitive domains assessed were identification (attention), detection (processing speed), Groton Maze Learning (executive function), International Shopping List (verbal learning), and One-Back (working memory). These particular tests reflect a subset of the consensus assessments that are thought to best capture CIAS and were selected based on their importance in schizophrenia (e.g., processing speed is thought to be a core deficit in schizophrenia) and the mechanism of action of KarXT [28]. Detailed descriptions of these tasks are provided in the appendix. The CBB was completed by patients at screening (to account for learning effects and ensure stable baseline measurements prior to active dosing), baseline, and week 5.

The PANSS [29] consists of 30 items, each scored on a scale from 1 to 7 (total score range 30‒210); higher scores represent greater severity of schizophrenia symptoms. The PANSS was administered at screening, baseline, and weeks 2, 4, and 5.

### Calculation of *Z*-scores and CBB composite

For each patient and CBB subtest score, a normative score indexed to healthy control performance (i.e., *Z*-score) was computed using the mean and SD from age-group‒stratified normative data (age categories 18–34 years, 35–49 years, 50–59 years, and 60–69 years) to index impairments relative to population norms. *Z*-scores were sign-corrected as needed to account for differences in whether higher or lower scores reflected poorer performance. A CBB composite score was created for each patient by computing the average (mean) across subtest *Z*-scores to provide a simplified overview measure that does not exclude potentially important variables over selecting a single cognitive domain. This approach also reduces the likelihood of findings that are associated with multiple comparisons across several individual cognitive tests and may be difficult to reliably interpret. The use of *Z*-score methods vs. alternative methods is supported by the greater interpretability of the measure compared with other methods (e.g., factor/principal component analysis) due to the method of standardising each subscale before inclusion and the equal contribution of each subscale to the overall composite scale.

### Classification of impaired and minimally impaired participants

Evaluable patients were separated into two groups based on cognitive impairment at study baseline using a CBB composite *Z*-score cutoff of –1 SD, relative to the normative mean. This cutoff was selected based on its previous use to differentiate clinically significant cognitive impairment from non-/minimal impairment in both patients with schizophrenia and more broadly in clinical neuropsychological assessment [30–32]. Patients with CBB composite *Z*-scores equal to or above this cutoff (*Z*-score ≥ –1) were designated as minimally impaired, whereas patients below the cutoff (*Z*-score < –1) were designated as clinically impaired.

### Calculation of intraindividual variability

To identify intraindividual variations in CBB scores that could reflect non-compliance or otherwise invalid data, intraindividual variability (IIV) across subtest performance was assessed for the evaluable sample by calculating the SDs across normatively corrected *Z*-scores within each subtest at baseline and end of study. The well-established 1.5 interquartile range (IQR) rule was used to define outlier IIV assessments as individual test scores falling outside quartile 3 + 1.5 IQR. The approach of identifying and removing patients who demonstrate highly variable assessment responses has been previously employed to examine treatment effects in mental illness, including schizophrenia and major depressive disorder [33, 34].

### Statistical analyses

The primary efficacy analysis for the trial was conducted using the modified intention-to-treat (mITT) population comprising all randomised patients who received at least one dose of study drug and had a valid baseline PANSS score and at least one post-baseline PANSS score. The current analysis included patients in the mITT population who completed the study and had data available on all subtests of the CBB to allow full characterisation of the cognitive profile and calculation of *Z*-scores and IIV (mITT completers).

Demographics and baseline characteristics of treatment (KarXT vs. placebo) and impairment (impaired vs. minimally impaired) subgroups were compared using analysis of variance and Chi-squared tests for continuous and categorical variables, respectively. Impact of IIV exclusion on sample characteristics, including performance differences on subtasks and change from baseline, were also compared using the same approach.

To explore the impact of patient cognitive impairment on response to KarXT, the analysis of covariance (ANCOVA) models were repeated on the impaired and minimally impaired groups separately. In addition, the impact of extreme variability in cognitive subdomain performance on treatment response was evaluated by applying the same models to the IIV-excluded sample. All statistical analyses were performed using SAS for Windows, Release 9.4 (SAS Institute, Cary, NC, USA).

Simple linear regression assessed the relationship between cognitive performance and PANSS scores at baseline and between the cognitive performance change from baseline to week 5 and PANSS total scores. ANCOVA models were used to estimate the effect of treatment on cognitive performance using the CBB composite score. Covariates included age, sex, site, and baseline CBB composite score. Least squares means change from baseline and differences from placebo were estimated along with SEs and 95% confidence intervals. Model-derived effect sizes (Cohen’s *d*) were also calculated.

## Results

### Characterisation of the patient population

The EMERGENT-1 patient population has been previously described [5]. Briefly, of the 182 patients enroled, 170 met the criteria for the mITT population. Of these 170 patients, 32 were subsequently excluded from the current analysis due to early termination or lack of valid PANSS scores at week 5. An additional 13 were subsequently excluded because of missing cognitive data on at least one subtest of the CBB (three at baseline and ten at day 35). The remaining 125 patients (mITT completers; KarXT *n* = 60, placebo *n* = 65) were included for analysis and designated as impaired (*n* = 60; KarXT *n* = 23, placebo *n* = 37) or minimally impaired (*n* = 65; KarXT *n* = 37, placebo *n* = 28).

Following IIV calculation, ten subtest observations (four at baseline and six at end of study) across eight patients exceeded the IQR threshold. The average IIV at baseline across the entire sample was 1.3 ± 0.8, suggesting that within this sample, a patient’s cognitive performance across subtests could be expected to vary by 1.3 SD from their CBB composite score. In contrast, the IIV average of eight outliers with excessive IIV scores on one or both CBB administrations (upper limit of IQR + 1.5: IIV > 2.4 at baseline or IIV > 2.5 at week 5) was more than double that at 2.9 ± 1.8.

Baseline characteristics were similar across impairment and treatment groups, with no significant differences in demographics or PANSS scores observed (Table 1). Similarly, baseline parameters, including PANSS scores, did not significantly differ between final sample included (*n* = 117) and IIV excluded (*n* = 8) patients (mean included PANSS 96.0 ± 7.6, mean excluded PANSS 97.5 ± 13.2, *p* = 0.60; Table 1). Characteristics for impairment groups further split by treatment are described in Supplementary Table S1 and Supplementary Table S2.

**Table 1:** Baseline characteristics by treatment, impairment, and IIV outlier subgroups.

	mITT completers by treatment			mITT completers by impairment level			IIV outliers		
	KarXT (*n* = 60)	Placebo (*n* = 65)	KarXT vs. placebo (*p* value)	Impaired (*n* = 60)	Minimally impaired (*n* = 65)	Impaired vs. minimally impaired (*p* value)	Included (*n* = 117)	Excluded (*n* = 8)	Included vs. excluded (*p* value)
Age, years	45.0 (10.3)	42.5 (9.8)	0.17	43.5 (9.8)	43.8 (10.4)	0.87	43.7 (10.8)	43.8 (10.9)	0.99
Sex, male, n (%)	48 (80.0)	45 (69.2)	0.24	42 (70.0)	46 (70.8)	0.38	87 (74.4)	6 (75.0)	1.00
PANSS total	96.4 (8.8)	95.7 (7.3)	0.15	96.9 (8.8)	95.3 (7.2)	0.25	96.0 (7.6)	97.5 (13.2)	0.60
PANSS negative	22.7 (4.4)	22.5 (4.2)	0.64	23.05 (4.64)	22.2 (3.9)	0.27	22.4 (4.3)	25.3 (3.6)	0.07
PANSS positive	25.8 (3.3)	26.1 (3.5)	0.67	26.1 (3.5)	25.8 (3.3)	0.60	26.0 (3.3)	25.1 (4.5)	0.47
CBB composite	–1.00 (1.00)	–1.25 (0.96)	0.85	–1.90 (0.83)	–0.42 (0.43)	<0.0001	–0.41 (0.49)	–2.25 (1.81)	0.0008

All values reflect group mean (standard deviation) unless otherwise indicated. CBB composite and subscale scores reflect normalised *Z*-score values. The *p* values reflect ANOVA-derived values for continuous variables and Chi-squared–derived values for factor or proportional variables. *ANOVA* analysis of variance, *CBB* Cogstate Brief Battery, *IIV* intraindividual variability, *mITT* modified intent to treat, *PANSS* Positive and Negative Syndrome Scale.

### KarXT treatment effect on cognitive performance by baseline impairment

In the impaired subgroup, KarXT was associated with statistically significant and meaningful (medium-large effect size) improvement in cognitive performance, both relative to baseline (LS means change from baseline 0.57, SE 0.19, *t* = 2.93, *p* = 0.01, *d* = 0.61) and compared with placebo (LS means change from baseline, difference from placebo 0.50, SE 0.22, *t* = 2.19, *p* = 0.03, *d* = 0.50; Table 2). No meaningful change in cognitive performance was observed in the placebo arm relative to baseline (LS means change from baseline 0.07, SE 0.13, *t* = 2.93, *p* = 0.59, *d* = 0.09; Table 2) in the impaired subgroup. In contrast, in the minimally impaired subgroup, there was no significant difference in cognitive performance between KarXT and placebo after 5 weeks of treatment (LS means change from baseline 0.04, SE 0.16, *t* = 0.55, *p* = 0.79, *d* = 0.05) nor significant change in performance in either arm across 5 weeks of treatment (see Table 2).

**Table 2:** KarXT treatment effect on cognitive performance by baseline impairment subgroup.

Sample	LS means change from baseline at day 35		95% confidence interval			
	Treatment	Estimate (SE)	Lower	Upper	*p* value	Cohen’s *d*
Minimally impaired	KarXT (*n* = 37)	–0.18 (0.13)	–0.44	0.09	0.19	0.22
	Placebo (*n* = 28)	–0.22 (0.15)	–0.52	0.08	0.15	0.28
	KarXT vs. placebo	0.04 (0.16)	–0.28	0.37	0.79	0.05
Impaired	KarXT (*n* = 23)	0.57 (0.19)	0.18	0.95	0.01	0.61
	Placebo (*n* = 37)	0.07 (0.13)	–0.19	0.33	0.59	0.09
	KarXT vs. placebo	0.50 (0.22)	0.04	0.95	0.03	0.50

LS means and *p* values are derived from post hoc ANCOVA models described earlier, with covariates of site, gender, age, and baseline performance. *ANCOVA* analysis of covariance, *LS* least squares.

### KarXT treatment effect on cognitive performance before and after removal of outliers

Prior to removal of IIV outliers, the change from baseline in CBB composite score was greater with KarXT than placebo, but the difference between treatment groups was not statistically significant (LS means change from baseline, difference from placebo 0.18, SE 0.13, *t* = 1.40, *p* = 0.16, *d* = 0.20; Table 3). Following outlier removal, a statistically significant overall treatment effect of KarXT vs. placebo was observed in CBB composite measure (KarXT *n* = 54, placebo *n* = 63, LS means change from baseline 0.27, SE 0.13, *t* = 2.05, *p* = 0.04, *d* = 0.31; Table 3).

**Table 3:** KarXT treatment effect on cognitive performance with and without IIV-derived outliers.

Sample	LS means change from baseline at day 35		95% confidence interval			
	Treatment	Estimate (SE)	Lower	Upper	*p* value	Cohen’s *d*
mITT completers	KarXT (*n* = 60)	0.13 (0.11)	–0.10	0.35	0.26	0.15
	Placebo (*n* = 65)	–0.05 (0.11)	–0.27	0.17	0.63	0.06
	KarXT vs. placebo	0.18 (0.13)	–0.08	0.44	0.16	0.20
mITT completers (IIV outliers excluded)	KarXT (*n* = 54)	0.22 (0.12)	–0.02	0.45	0.07	0.25
	Placebo (*n* = 63)	–0.05 (0.11)	–0.27	0.17	0.63	0.06
	KarXT vs. placebo	0.27 (0.13)	0.01	0.53	0.04	0.31

LS means and *p* values are derived from post hoc ANCOVA models described earlier, with covariates of site, gender, age, and baseline performance. *ANCOVA* analysis of covariance, *IIV* intraindividual variability, *LS* least squares, *mITT* modified intention to treat, *SE* standard error.

### KarXT treatment effect on cognitive performance by baseline impairment before and after removal of outliers

After the removal of IIV-derived outliers (minimally impaired: KarXT, *n* = 3; placebo, *n* = 0; impaired: KarXT, *n* = 3; placebo, *n* = 2) in the impaired subgroup, KarXT was again associated with statistically significant improvement in cognitive performance compared with baseline (LS means change from baseline 0.66, SE 0.18, *t* = 3.49, *p* < 0.01, *d* = 0.53), and relative to placebo (LS means change from baseline, difference from placebo 0.6, SE 0.22, *t* = 2.73, *p* < 0.01, *d* = 0.79; Table 4). Further, the effect size was larger (at both the treatment- and group-comparison levels) than when IIV-derived outliers were not removed. No meaningful change in cognitive performance was observed in the placebo arm compared with baseline (LS means change from baseline 0.06, SE 0.12, *t* = 0.46, *p* = 0.65, *d* = 0.08; Table 4) in the impaired subgroup. In contrast, in the minimally impaired subgroup, there was no significant difference in cognitive performance between KarXT and placebo after 5 weeks of treatment (LS means change from baseline, difference from placebo 0.06, SE 0.17, *t* = 0.35, *p* = 0.73, *d* = 0.07; Table 4). Evidence suggesting regression to the mean (worsening of performance seen in both treatment arms) in the minimally impaired subgroup appeared to be driven primarily by IIV outliers.

**Table 4:** KarXT treatment effect on cognitive performance by baseline impairment subgroup, IIV outliers removed.

Sample	LS means change from baseline at day 35		95% confidence interval			
	Treatment	Estimate (SE)	Lower	Upper	*p* value	Cohen’s *d*
Minimally impaired	KarXT (*n* = 34)	–0.017 (0.15)	–0.32	0.28	0.9	0.02
	Placebo (*n* = 28)	–0.078 (0.15)	–0.38	0.22	0.6	0.09
	KarXT vs. placebo	0.06 (0.17)	–0.29	0.41	0.73	0.07
Impaired	KarXT (*n* = 20)	0.66 (0.18)	0.28	1.00	0.001	0.53
	Placebo (*n* = 35)	0.06 (0.12)	–0.19	0.30	0.65	0.08
	KarXT vs. placebo	0.6 (0.22)	0.16	1.10	0.009	0.79

LS means and *p* values are derived from post hoc ANCOVA models described earlier, with covariates of site, gender, age, and baseline performance. *ANCOVA* analysis of covariance, *IIV* intraindividual variability, *LS* least squares.

### Relationship between CBB composite scores and PANSS scores

**Fig. 1:**
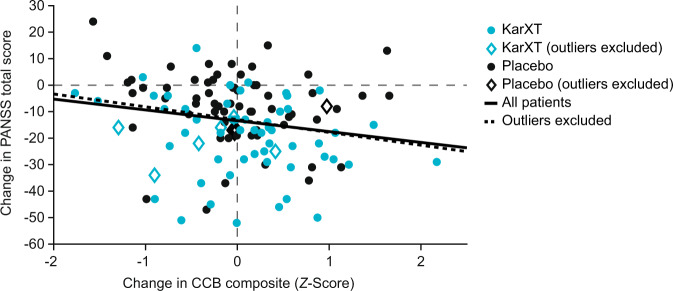
Relationship between CBB composite score change and PANSS total score change, between baseline and after 5 weeks of treatment. *CBB* Cogstate Brief Battery, *PANSS* Positive and Negative Syndrome Scale.

There was a small but statistically significant relationship between PANSS total and CBB composite scores at baseline (*β* = –0.19, *p* = 0.03, adjusted *R*^2^ = 0.07) and between change from baseline to week 5 in PANSS total and CBB composite scores (*β* = –0.20, *p* = 0.03, adjusted *R*^2^ = 0.03; Fig. 1) in the overall evaluable sample and when outliers were excluded (*β* = –0.22, *p* = 0.02, adjusted *R*^2^ = 0.04). Thus, the effect of KarXT on cognitive performance appears to be largely independent of KarXT’s antipsychotic effects.

## Discussion

The data presented here suggest that, in addition to significantly improving positive and negative symptoms of schizophrenia in the phase 2 EMERGENT-1 study [5], the M_1_/M_4_-preferring mAChR agonist KarXT shows promise as a treatment for CIAS. In initial analyses of the exploratory cognitive endpoint in the phase 2 EMERGENT-1 study, KarXT showed a trend towards greater improvement in cognitive performance relative to placebo over the course of 5 weeks of treatment but did not reach statistical significance. However, more than half of the study participants did not show clinically meaningful cognitive impairment compared with normative standards at baseline in our sample. In this post hoc analysis of only patients who performed at or lower than –1 SD below the mean level of performance for healthy controls (clinically impaired), a significant treatment effect was observed for KarXT compared with placebo. We believe this suggests that KarXT may be a potentially beneficial treatment for CIAS among patients who exhibit prototypical levels of cognitive impairment and are thus able to benefit from treatment.

Additionally, a few extreme outliers in performance on the cognitive outcomes measures had an outsized impact on the analysis by increasing overall variability. When excluding only eight patients with patterns of extreme variability between subtests, which are highly unlikely to be reflective of true cognitive ability and may be due to task non-compliance, we showed a significant treatment effect for KarXT vs. placebo across the entire sample regardless of baseline cognitive performance. This finding is particularly notable given the number of patients in the sample who were performing in the normative range cognitively at baseline.

Although these post hoc findings must be replicated in future studies, they are particularly promising for two reasons. First, neither the trial design nor the cognitive endpoint was chosen to maximise the probability of detecting cognitive change. In such a short trial (5 weeks) with acutely psychotic patients who may struggle to complete computerised cognitive assessments, the consistent results favouring KarXT are potentially indicative of a unique effect on cognition, particularly given the relative independence of treatment effects in cognition and psychosis. Second, KarXT showed significant benefit vs. placebo when patients with either minimal baseline impairment or excessive performance variability were excluded, consistent with other placebo-controlled trials of xanomeline in patients with AD [21] and schizophrenia [22]. Limiting study inclusion to patients with schizophrenia exhibiting consistent cognitive impairment, or prespecifying such analyses, should be considered in future trials.

Studies of antidepressants and antipsychotics suggest that patients with minimal baseline symptom severity are less likely to show meaningful differences between drug and placebo, likely due to ceiling effects [35, 36]. Although post hoc analyses must always be viewed in the appropriate context, stratifying based on cognitive impairment at baseline may be a useful strategy to account for the heterogeneity in cognitive performance among patients with schizophrenia, thus accounting for ceiling effects that may obscure benefits of treatment in specific patient subsets.

Between one in four and one in three patients with schizophrenia fall within the normative range of cognitive performance compared with age-matched controls [1]. In the current study, the inclusion of a large number of patients with minimal cognitive impairment likely negatively impacted our ability to detect therapeutic changes sample-wide. Further, patients recruited for complex and lengthy trials are likely to have disproportionately lower levels of cognitive impairment than the population of people with schizophrenia, or those recruited specifically for CIAS trials. Although all patients with schizophrenia may exhibit some cognitive decline relative to premorbid functioning, validly characterising and controlling for premorbid cognitive function is not feasible in clinical trial designs because of the challenges in obtaining information on premorbid function. As a result, among patients performing at or near the normative range, it is not possible to identify those with a decline in cognitive performance from those who may be cognitively intact. The inclusion of such patients, particularly if they represent a substantial portion of the overall sample, may reduce the ability to detect therapeutic change. This is particularly true given that it is unclear if reversing cognitive decline vs. augmenting normal performance would both arise from the same treatment strategy. For example, there is reason to believe the drugs that target the cholinergic system (e.g., acetylcholinesterase inhibitors) may only be beneficial for those experiencing cognitive impairment [37].

In this analysis, we stratified patients into two groups using a cutoff of –1 SD relative to the average performance level of healthy controls using our cognitive test battery. This threshold is recommended for use in clinical neuropsychological assessments to distinguish between clinically impaired and unimpaired patients, and has been used previously to differentiate patients with schizophrenia with relatively intact cognitive function from those with typical levels of cognitive impairment [32]. The cognitive trajectories and treatment responses of these two groups differ significantly. For example, patients who exhibit minimal to no cognitive impairment have been shown to respond differentially to both pharmacological and behavioural treatments for CIAS. Higher baseline cognitive performance has been associated with reduced gains in cognitive training [31, 38], with different findings in extremely chronic and currently institutionalised patients [39]. Additionally, differing relationships with functional outcomes in these two groups have been observed. Whereas there is no relationship between cognitive impairment and everyday functioning in patients who score 40 points or higher (*t*-score; equivalent to –1 SD below the normal range), greater cognitive impairment is associated with greater functional impacts in patients who score less than 40 [40]. Thus, although CIAS is often defined as ubiquitous in patients with schizophrenia (i.e., there is no established minimal level of impairment that defines CIAS), we believe this cutoff score captures a population of interest: those who are currently exhibiting at least modest cognitive impairment and therefore most likely to derive therapeutic benefit. However, it is worthwhile to note that this cutoff score is still relatively conservative, as even in clinical trials sample composite cognitive performance is commonly found to be 2.0 or more SD below normative standards [41].

In our sample, patients with minimal impairment at baseline who received KarXT or placebo did not exhibit significant changes in cognitive performance over 5 weeks of treatment (Table 2). Although minimally impaired patients in both arms exhibited some worsening in cognitive performance, this effect was not statistically significant and appeared to be driven largely by IIV outliers, as the effect was substantially lessened upon their exclusion (Table 4). In contrast, there was a meaningful and significant effect of KarXT on cognition in impaired patients, whereas patients in the impaired group receiving placebo showed no meaningful change in performance. The magnitude of improvement in impaired patients is consistent with the level of change designated as clinically meaningful by the American Psychiatric Association, in their discussion about cognitive training as a standard of care intervention in schizophrenia [42]. Notably, 23 of 60 patients (38.3%) in the KarXT group were cognitively impaired at baseline compared with 37 of 65 (56.9%) of those in the placebo group. The chance overrepresentation of cognitively impaired patients in the placebo group may have further biased results in the mITT population towards the null hypothesis.

In a separate analysis, we also examined the effect of removing outlier test administrations that were highly variable. Excessive variability reduces the power to detect meaningful change and can bias results towards the null. For example, patients who exhibit large changes in cognitive performance across pretreatment timepoints have been shown to have larger placebo responses and would therefore be likely to contribute to smaller drug-placebo differences [26]. Previous studies in patients with schizophrenia suggest that increased IIV in performance across cognitive subdomains is higher in this group compared with healthy controls and may be a marker of overall greater cognitive impairment [43, 44]. However, IIV in patients with schizophrenia typically does not exceed 1 SD on average [43]. Variability that is in excess of what could reasonably be expected to reflect true cognitive performance may be a useful marker of administration error or patient task non-engagement [33].

In a post hoc analysis of IIV across subtests within the same CBB administration, IIV was approximately 1.3, with 76% of patients having an average IIV of less than 1.5. In contrast, the average IIV of patients who were excluded for excessive between-test variability was 2.9 at baseline and 3.1 at end of study. The cognitive profile of the excluded patients was highly irregular and unlikely to represent true cognitive performance. Following outlier removal, a significant overall treatment effect of KarXT (*n* = 54) vs. placebo (*n* = 63) was observed (estimate [SE] 0.27 [0.13], *t* = 2.05, *p* = 0.04, *d* = 0.31). Baseline PANSS scores did not significantly differ between included and excluded patients (Table 1), suggesting that the presence of positive or negative symptoms is an unlikely causal driver of highly variable cognitive performance.

Flagging and potentially censoring assessments in which lack of correlation between domains of cognitive function that are believed to be interrelated is not a novel approach for endpoints. For example, item-level consistency checks are frequently used when administering the PANSS to either alert the interviewer or flag potentially problematic data for intervention [45]. In a post hoc analysis of a phase 2 study in schizophrenia, Kott and colleagues (2021) demonstrated that erratic ratings or extreme changes in scores across assessment points (i.e., extremely variable PANSS scores) were associated with reduced drug-placebo difference [33]. The approach taken in the current study could be implemented in a similar but prespecified fashion in future CIAS studies, using extreme variability across subtest performance in combination with other stability criteria (e.g., a limit on change in performance across baseline and screening assessments) to exclude patients prospectively. Although CBB assessments with extreme IIV were excluded at all timepoints in our post hoc analysis, this approach could be adapted as a screening tool. Future studies may consider implementing screening criteria designed to exclude patients who are either unable or unwilling to validly engage in CBB assessments.

The current study had limitations inherent to post hoc analyses. The primary focus of the study was on the effect of KarXT on psychotic symptoms, so CBB scores were not available at endpoint for patients terminating early. In addition, the small number of cognitive subtests and the use of IIV as an outlier measure precluded inclusion of participants who had invalid or missing scores for one or more of the subtests. The resulting potential sample bias and small sample size is a significant limitation on the interpretation of the cognition data. Among patients in the minimally impaired group, there was a slight worsening in performance between baseline and end of study. However, these effects were not significant and appear to be driven by patients who were excluded for extremely variable performance (high IIV). When excluding these patients, the change in cognitive performance in the placebo or KarXT arms in the minimally impaired subgroup was very small (<0.1 standard score). Furthermore, no meaningful nor significant change in performance was observed in the placebo arm among the impaired subgroup in either analysis, making it unlikely that regression to the mean would account for the findings in the other group. The CBB was completed both at screening and baseline to account for learning or practice effects, but considering the repeated testing on multiple visits, practice effects cannot be excluded. However, given the small and statistically non-significant changes from baseline in CBB composite scores in the placebo arm across all evaluated populations, a practice effect accounting for the drug effect is unlikely. Correlations between improvements in CBB score and PANSS total, although statistically significant, were modest. Improvement in positive and negative symptoms of schizophrenia accounted for less than 3% of the total variance in cognitive performance. The small amount of shared variance suggests that KarXT may have independent impacts on both cognitive functioning and other symptoms of schizophrenia. These findings may not generalise to other cognitive batteries. However, studies with other assessment batteries, such as the Brief Assessment of Cognition in Schizophrenia, Cambridge Neuropsychological Test Automated Battery, and MATRICS™ Consensus Cognitive Battery, have shown similar effects to those reported here, including in successful cognitive training studies [26, 30].

The results of the post hoc analyses presented here provide insight into the potential impact of KarXT on cognitive performance in patients with schizophrenia participating in the phase 2 EMERGENT-1 acute psychosis trial. Although initial analyses of the exploratory cognitive endpoint showed a small effect for KarXT on cognitive performance that was not statistically significant vs. placebo, the alternative analyses presented here detected a clinically meaningful and statistically significant benefit of KarXT vs. placebo. The application of a minimal symptom severity or stability criterion is common practice in other therapeutic areas and in studies of positive and negative symptoms of schizophrenia. Application of these approaches in prospective statistical analysis plans or predefined study entry criteria seems warranted. Future studies are necessary to replicate and expand upon these findings to further characterise the potential effects of KarXT on cognition in patients with schizophrenia.

## Supplementary information

Detailed Description of the Cogstate Brief Battery (CBB)
