# Supplementary material for: Effectiveness of KarXT (xanomeline-trospium) for cognitive impairment in schizophrenia: post hoc analyses from a randomised, double-blind, placebo-controlled phase 2 study
Source: Transl Psychiatry. 2022 Nov 21;12:491. doi: 10.1038/s41398-022-02254-9 (PMC9681874; doi:10.1038/s41398-022-02254-9)
Supplement: Supplementary file 1 — Detailed Description of the Cogstate Brief Battery (CBB) [file 41398_2022_2254_MOESM1_ESM.pdf]

SUPPLEMENTARY INFORMATION

Appendix 1

Detailed Description of the Cogstate Brief Battery (CBB)

The CBB was administered to study participants to assess the core domains of cognitive impairment associated with schizophrenia in a low-burden manner. The CBB includes five computerised cognitive tests that are completed on an iPad. The tasks and cognitive domains assessed, including a brief description, were:

- Identification (IDN). The IDN task is an assessment of attention in which participants must determine as quickly as possible whether the colour of a playing card presented to them is red or not. The onscreen instruction question reads, “Is the card red?” On each trial, the card is initially faced down but is then flipped over and revealed to the participant who must then press “Yes” if the card is red or “No” if it is not. Participants are encouraged to be as quick and accurate as possible. The key outcome measure is speed of performance.
- Detection (DET). DET assesses psychomotor function/speed using a simple reaction time paradigm. Similar to IDN, a playing card is initially faced down. This time, the onscreen instruction question is “Has the card turned over?” As soon as the card turns over, the participant must press “Yes” as quickly as possible. The key outcome measure is speed of performance.
- Groton Maze Learning Test (GMLT). The GMLT assesses executive function using a maze-learning paradigm. A 10x10 grid of tiles is presented to the participant on the screen, among which a 28-step pathway is hidden. A blue tile indicates the start and a tile with red circles

indicates the finish. The participant must move one step at a time from the start towards the end by touching a tile next to their current location. If the correct move is made, a green check mark appears and if the move is incorrect a red cross is revealed. Once completed, the participant returns to the start location to repeat the test and must try to remember the pathway just completed. The key outcome measure is the total number of errors made when attempting to learn the same hidden pathway on five consecutive trials during a single session.

- International Shopping List (ISL). The ISL task is a well-established and commonly deployed assessment of verbal learning that utilises a list-learning paradigm. The participants must read a shopping list and remember and recall as many items on the list as possible. The key outcome measure is the total number of correct responses made in remembering the list on three consecutive trials during a single session.
- One-Back (ONB). The ONB task assesses working memory using the n-back paradigm. Another task utilising playing cards, the ONB's onscreen instruction asks, "Is the previous card the same?" A playing card is presented face up in the centre of the screen. The participant must decide whether the card is the same as the previous card. If the card is the same, the participant should press "Yes." If the card is not the same, the participant should press "No." The participant is encouraged to work as quickly and accurately as possible.

Table S1. Baseline demographics and clinical characteristics for impairment groups by treatment.

	**Minimally impaired (*n* = 65)**		**Impaired (*n* = 60)**	
	**KarXT (*n* = 37)**	**Placebo (*n* = 28)**	**KarXT (*n* = 23)**	**Placebo (*n* = 37)**
Age, years	45.00 (10.81)	42.29 (9.81)	44.91 (9.56)	42.68 (9.95)
Sex, male, n (%)	28 (75.7)	22 (78.6)	19 (82.6)	23 (62.2)
PANSS total	95.86 (7.91)	94.46 (6.14)	97.26 (10.16)	96.68 (7.95)
PANSS negative	22.14 (3.07)	22.29 (4.81)	23.57 (5.86)	22.73 (3.74)
PANSS positive	25.95 (2.98)	25.64 (3.66)	25.65 (3.88)	26.43 (3.29)
CBB composite (*Z*-score)	–0.41 (0.49)	–0.45 (0.36)	–1.95 (0.86)	–1.86 (0.81)
Identification	–0.04 (1.28)	–0.35 (1.07)	–1.71 (1.41)	–2.49 (1.24)
Detection	0.03 (1.27)	–0.48 (1.29)	–1.47 (1.65)	–1.84 (1.30)
Groton Maze Learning	–0.86 (1.40)	–0.64 (1.23)	–2.58 (2.27)	–2.43 (3.05)
One-Back	–0.44 (1.10)	–0.21 (1.23)	–2.48 (1.57)	–1.34 (1.48)
International Shopping List	–0.72 (0.91)	–0.55 (0.84)	–1.53 (0.80)	–1.22 (0.86)

All values reflect group means (standard deviation) unless otherwise indicated. *CCB* Cogstate Brief Battery, *PANSS* Positive and Negative Syndrome Scale.

Table S2. Baseline Cogstate Brief Battery subtest scores by treatment, impairment, and IIV outlier subgroups.

	**mITT completers by treatment**			**mITT completers by impairment level**			**IIV outliers**		
	**KarXT (*n* = 60)**	**Placebo (*n* = 65)**	**KarXT vs. placebo (*p* value)**	**Impaired (*n* = 60)**	**Minimally impaired (*n* = 65)**	**Impaired vs. minimally impaired (*p* value)**	**Included (*n* = 117)**	**Excluded (*n* = 8)**	**Included vs. excluded (*p* value)**
Identification	–0.68 (1.55)	– 1.57 (1.57)	0.002	–2.19 (1.35)	–0.17 (1.19)	<0.0001	–1.13 (1.55)	–1.25 (2.54)	0.83
Detection	–0.55 (1.59)	–1.26 (1.45)	0.010	–1.70 (1.44)	–0.19 (1.29)	<0.0001	–0.90 (1.46)	–1.19 (2.74)	0.61
Groton Maze Learning	–1.52 (1.96)	–1.66 (2.58)	0.75	–2.49 (2.75)	–0.77 (1.33)	<0.0001	–1.29 (1.58)	–6.03 (5.27)	<0.0001
One-Back Accuracy	–1.22 (1.63)	–0.85 (1.48)	0.19	–1.77 (1.60)	–0.34 (1.15)	<0.0001	–0.97 (1.49)	–1.82 (2.29)	0.14
International Shopping List	–1.03 (0.95)	–0.93 (0.91)	0.55	–1.34 (0.84)	–0.65 (0.88)	<0.0001	–0.98 (0.91)	–0.93 (1.23)	0.88

All values reflect group mean (standard deviation) unless otherwise indicated. CBB composite and subscale scores reflect normalised *Z*-score values. The *p* values reflect ANOVA-derived values for continuous variables and Chi-squared–derived values for factor or proportional variables. *ANOVA* analysis of variance, *CCB* Cogstate Brief Battery, *IIV* intraindividual variability, *mITT* modified intent to treat.
